# N-terminal Huntingtin Knock-In Mice: Implications of Removing the N-terminal Region of Huntingtin for Therapy

**DOI:** 10.1371/journal.pgen.1006083

**Published:** 2016-05-20

**Authors:** Xudong Liu, Chuan-En Wang, Yan Hong, Ting Zhao, Guohao Wang, Marta A. Gaertig, Miao Sun, Shihua Li, Xiao-Jiang Li

**Affiliations:** 1 State Key Laboratory of Molecular Developmental Biology, Institute of Genetics and Developmental Biology, Chinese Academy of Sciences, Beijing, China; 2 Department of Human Genetics, Emory University School of Medicine, Atlanta, Georgia, United States of America; 3 Graduate School of the Chinese Academy of Sciences, Beijing, China; Thomas Jefferson University, UNITED STATES

## Abstract

The Huntington’s disease (HD) protein, huntingtin (HTT), is a large protein consisting of 3144 amino acids and has conserved N-terminal sequences that are followed by a polyglutamine (polyQ) repeat. Loss of Htt is known to cause embryonic lethality in mice, whereas polyQ expansion leads to adult neuronal degeneration. Whether N-terminal HTT is essential for neuronal development or contributes only to late-onset neurodegeneration remains unknown. We established HTT knock-in mice (N160Q-KI) expressing the first 208 amino acids of HTT with 160Q, and they show age-dependent HTT aggregates in the brain and neurological phenotypes. Importantly, the N-terminal mutant HTT also preferentially accumulates in the striatum, the brain region most affected in HD, indicating the importance of N-terminal HTT in selective neuropathology. That said, homozygous N160Q-KI mice are also embryonic lethal, suggesting that N-terminal HTT alone is unable to support embryonic development. Using Htt knockout neurons, we found that loss of Htt selectively affects the survival of developing neuronal cells, but not astrocytes, in culture. This neuronal degeneration could be rescued by a truncated HTT lacking the first 237 amino acids, but not by N-terminal HTT (1–208 amino acids). Also, the rescue effect depends on the region in HTT known to be involved in intracellular trafficking. Thus, the N-terminal HTT region may not be essential for the survival of developing neurons, but when carrying a large polyQ repeat, can cause selective neuropathology. These findings imply a possible therapeutic benefit of removing the N-terminal region of HTT containing the polyQ repeat to treat the neurodegeneration in HD.

## Author Summary

The 17 amino acids in the N-terminal region of huntingtin (HTT) are conserved in a wide range of species and are followed by a polyglutamine repeat whose expansion causes selective neurodegeneration in Huntington’s disease (HD). Loss of Htt can affect developing neurons and early embryonic development in mice. Whether N-terminal HTT is important for the survival of developing neurons or contributes mainly to a gain of toxic function in HD remains unknown. In the current study, we generated N-terminal mutant HTT knock-in mice and found that N-terminal HTT with an expanded polyQ repeat is unable to support the early development of mice, but can cause age-dependent neurological phenotypes. Further, we show that a truncated HTT without the N-terminal region can rescue the Htt loss-mediated degeneration of developing neurons. Our studies suggest that removal of the N-terminal region of mutant HTT could be a strategy to abolish the neuronal toxicity of mutant HTT.

## Introduction

Huntington’s disease (HD) is caused by a polyglutamine expansion in the N-terminal region of huntingtin (HTT). Despite the protein’s ubiquitous expression in the brain and body, mutant HTT causes selective neuronal degeneration in the brain, which is characterized by the preferential loss of neuronal cells in the striatum in the early disease stage and extensive neurodegeneration in a variety of brain regions in later disease stages [1]. The progressive neurodegeneration is consistent with late-onset neurological symptoms in HD, which become increasingly severe with age and lead to death 10–15 years after the onset of symptoms. Thus, age-dependent mutant HTT toxicity is a characteristic of HD, and understanding the mechanism behind this toxicity is key to developing effective treatments for HD.

Normal HTT consists of 3144 amino acids and is considered to be a scaffold protein that associates with a number of other proteins and participates in a wide range of cellular functions, including intracellular trafficking of a variety of proteins [2, 3]. In support of HTT’s important function, knocking out the *Htt* gene leads to the early death of mouse embryos at embryonic day 8.5 [4–6]. Interestingly, mutant HTT with expanded polyglutamine can rescue this embryonic lethality phenotype [7,8], indicating that the expansion of polyglutamine does not affect the early development of the animal, but causes late-onset neurodegeneration and neurological symptoms. Also, the polyQ domain appears to be non-essential for low species. For example, the polyQ stretch is absent in the N-terminal of HTT in Drosophila melanogaster and Ciona intestinalis (sea squirt) and contains four glutamines in fish, birds, and amphibians [9]. The proline-rich domain (polyP), which follows the polyQ stretch, is found only in mammals. Although polyP may contribute to the solubility of HTT [10], deletion of polyQ or the proline-rich domain does not affect mouse development [11,12]. N-terminal HTT fragments can be generated by proteolytic processing and other mechanisms. For example, exon 1 HTT is produced by aberrant splicing of the HTT gene [13]. It is important to investigate whether N-terminal HTT is required for the early development or survival of mammalian animals.

Although evidence from a variety of HD mouse models indicates a toxic gain of function from polyglutamine expansion, important issues remain to be addressed. First, we know that HD mice expressing full-length HTT show preferential accumulation of N-terminal HTT aggregates in the mouse striatum [14–17], although whether this striatal accumulation requires the context of full-length HTT remains unclear. Another important issue is whether N-terminal HTT is essential for early embryonic or animal development. Addressing this issue is especially critical for understanding the function of HTT, since extensive studies have focused on the N-terminal 17 amino acids of HTT that are very conserved in various species [18–23].

Given the important issues above, we established a new HD mouse knock-in model that expresses N-terminal mutant HTT (208 amino acids) with 160Q under the control of the endogenous mouse promoter. These heterozygous mice show a preferential accumulation of mutant HTT in striatal neurons and age-dependent neurological phenotypes. Moreover, expression of N-terminal HTT cannot rescue the embryonic lethal phenotypes in the absence of full-length mouse Htt. Rather, we found that HTT lacking the N-terminal region (1–237 amino acids) was able to rescue the degeneration of cultured neurons due to the loss of Htt. Our studies suggest that the N-terminal region of HTT is nonessential for developing neurons or neuronal survival, and therefore can be removed to eliminate polyQ toxicity in treating HD.

## Results

### Generation of N-terminal HTT knock-in mice

**Fig 1:**
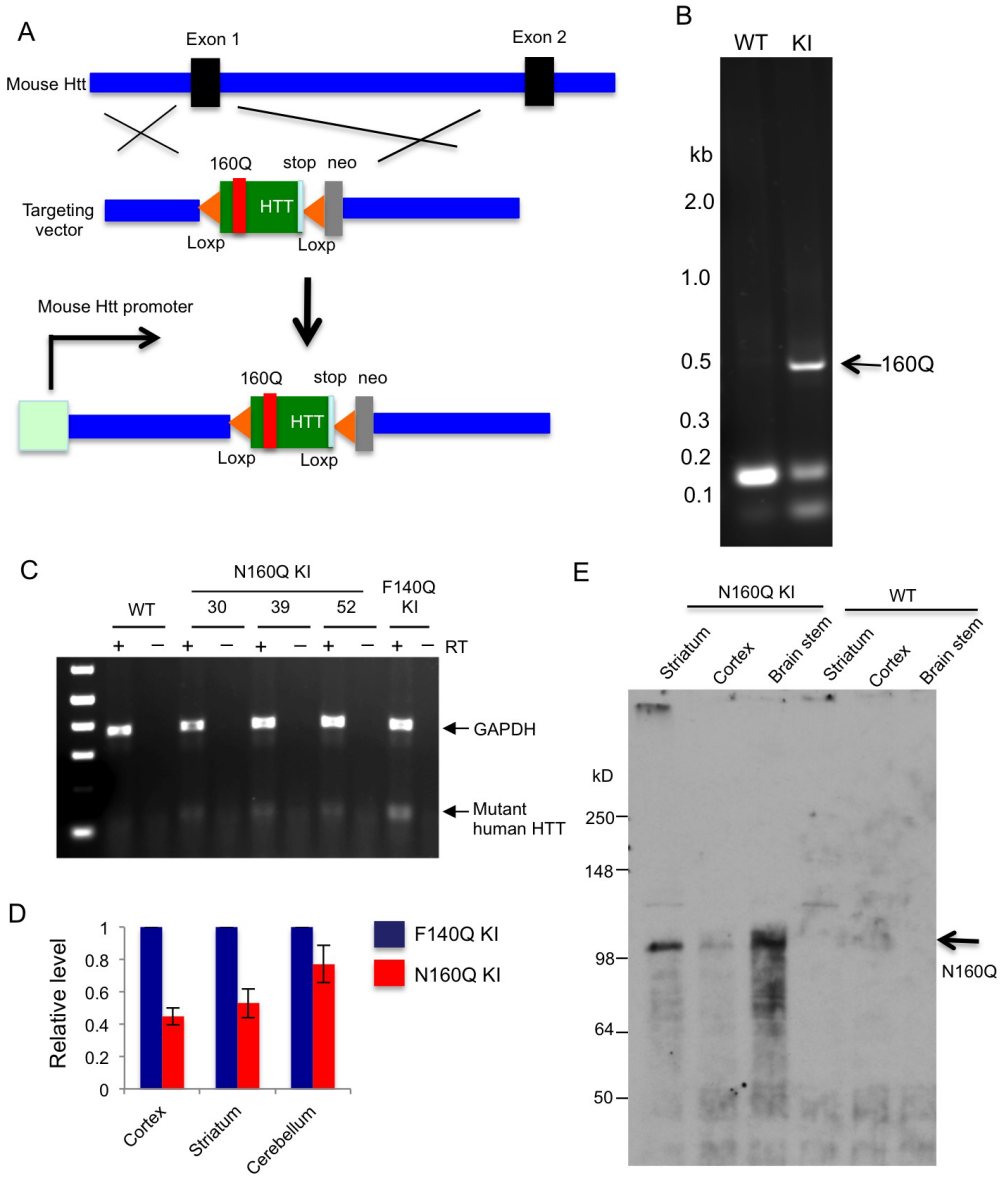
Generation of N-terminal HTT knock-in mice. (**A**) Structure of targeting vectors used for generating N160Q KI mice. Human *HTT* cDNA encoding the first 208 amino acids plus 160Q with a stop codon is inserted into the mouse *Htt* exon1. Neo selection marker is inserted into the intron after exon1 of the mouse HD gene. Two loxP sites also flank N-terminal HTT and neo cDNAs. (**B**) Genotyping of 160Q KI mouse tails showing the increased CAG repeat for 160Q. (**C**) RT-PCR analysis of N160Q KI mice (#30, #39, and #52). Primers specific to human *HTT* were used for PCR. WT and heterozygous full-length mutant HTT KI (F140Q KI) mice served as controls. Reverse transcripts (RT) were added or excluded in PCR reactions. (**D**) Quantitative RT-PCR shows the relative levels of N-terminal mutant HTT transcripts. The samples were obtained from the cortex, striatum, and cerebellum from heterozygous N160Q KI and F140Q KI mice. The relative levels of mutant HTT in N160Q KI mice were normalized by GAPDH levels and compared with full-length mutant HTT in heterozygous F140Q KI mice. (**E**) Expression of N-terminal mutant HTT in KI mouse brains via western blotting analysis. Arrows indicated N-terminal mutant HTT. The blot was probed with 1C2 antibody at 1:4000 dilution.

To investigate the function and toxicity of N-terminal mutant HTT, we replaced exon1 of the mouse *Htt* gene with the cDNA encoding the first 208 amino acids of human HTT containing 160Q (N160Q) and a stop codon via gene targeting in mouse embryonic stem (ES) cells (Fig 1A). The targeting vector containing two loxP sites that flank the human *HTT* DNAs and neomycin resistant gene was transfected into mouse ES cells. Positive ES cells containing the targeted HD gene were then injected into C57B1/6J blastocysts to generate heterozygous N160Q KI mice. PCR and DNA sequencing analyses of genomic DNA verified the presence of the large CAG repeat encoding 160Q (Fig 1B). Comparing heterozygous N160Q KI with heterozygous full-length HTT (F140Q) KI mice via RT-PCR analysis of *HTT* mRNA expression indicated that N160Q is expressed at a lower level than full-length mutant HTT in F140Q KI mice (Fig 1C). Quantitative analysis of mRNA expression suggests that N160Q is expressed at 51–75% of full-length mutant HTT in different brain regions (Fig 1D). The insertion of the Neo selection DNA in the intron after exon1 may interfere with splicing or RNA stability, resulting in a lower level of mutant *HTT* mRNAs than endogenous normal *HTT* mRNAs. The expression of N160Q at the protein level in the mouse brain is confirmed by western blotting with 1C2 antibody that specifically reacts with the expanded polyQ repeats in N-terminal mutant HTT (Fig 1E).

### Age-dependent accumulation of N160Q in the striatum and reactive astrocytes

**Fig 2:**
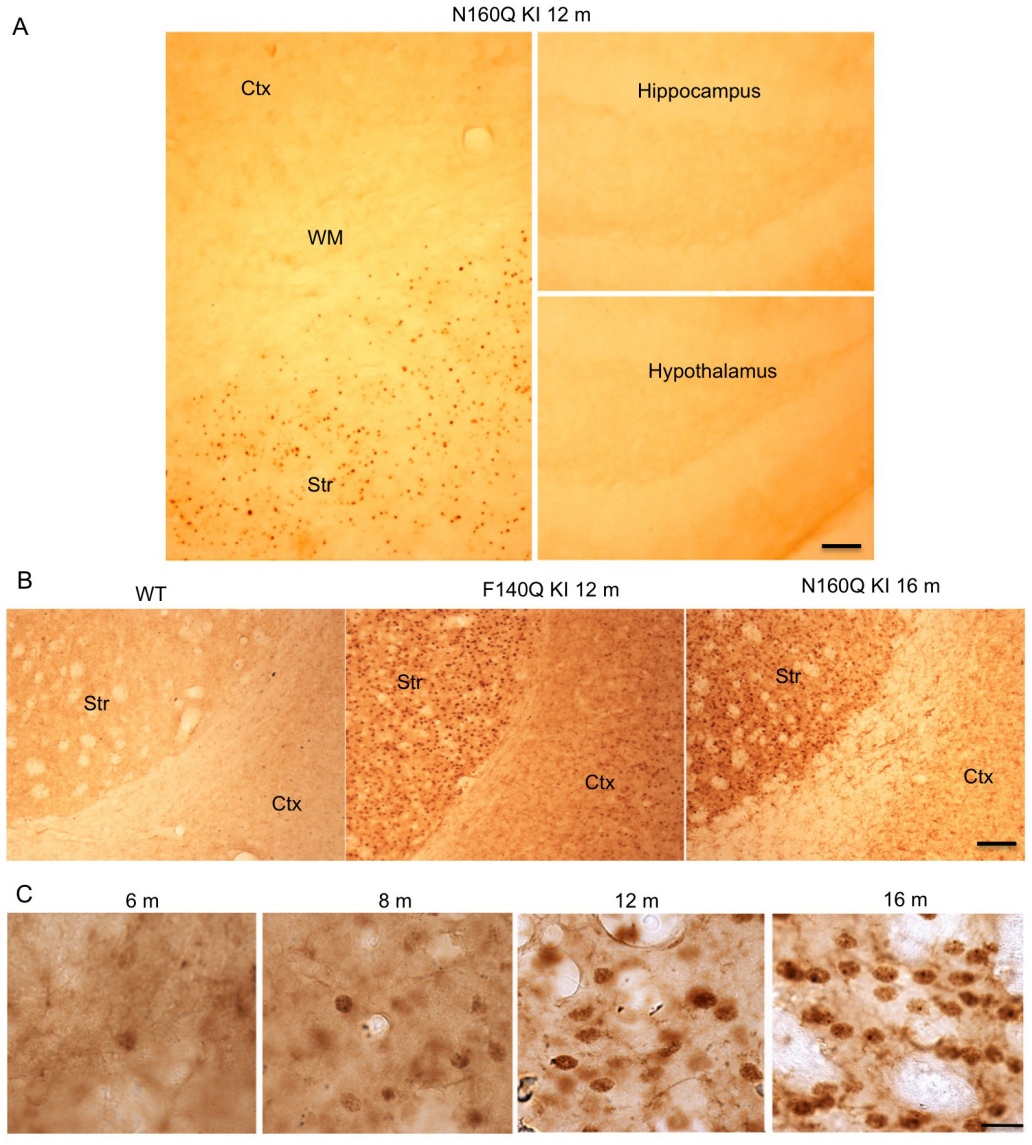
Preferential accumulation of N-terminal mutant HTT in the striatum in KI mice. (**A**) EM48 staining showing the more abundant distribution of mutant HTT in the striatum than the cortex in N160Q KI mice at 12 months of age. (**B**) EM48 staining also demonstrates the preferential distribution of mutant HTT in the striatum in full-length HTT KI (N140Q KI) and N160Q KI mice. (**C**) Age-dependent increases in HTT accumulation in N160Q mice at 6, 8, 12, and 16 months of age. Scale bar: (A and B): 100 μm; (C): 10 μm.

We used 1C2 immunostaining to examine the distribution of N160Q in KI mice and found that N160Q KI mice at 1 year of age show obvious HTT staining in the striatum, which is more abundant than in other brain regions, such as the cortex and cerebellum (Fig 2A). This preferential accumulation of N160Q in the striatum is more abundant in mice at the age of 16 months and is similar to F140Q KI mice that express full-length mutant HTT at 12 months (Fig 2B). Like F140Q and other KI mice in which mutant HTT accumulates in the striatum in an age-dependent manner [14–17], N160Q-KI mice show a progressive accumulation of mutant HTT in the striatum (Fig 2C). Thus, the first 208 amino acids of HTT appear to be capable of mediating the preferential distribution of HTT in the striatum.

**Fig 3:**
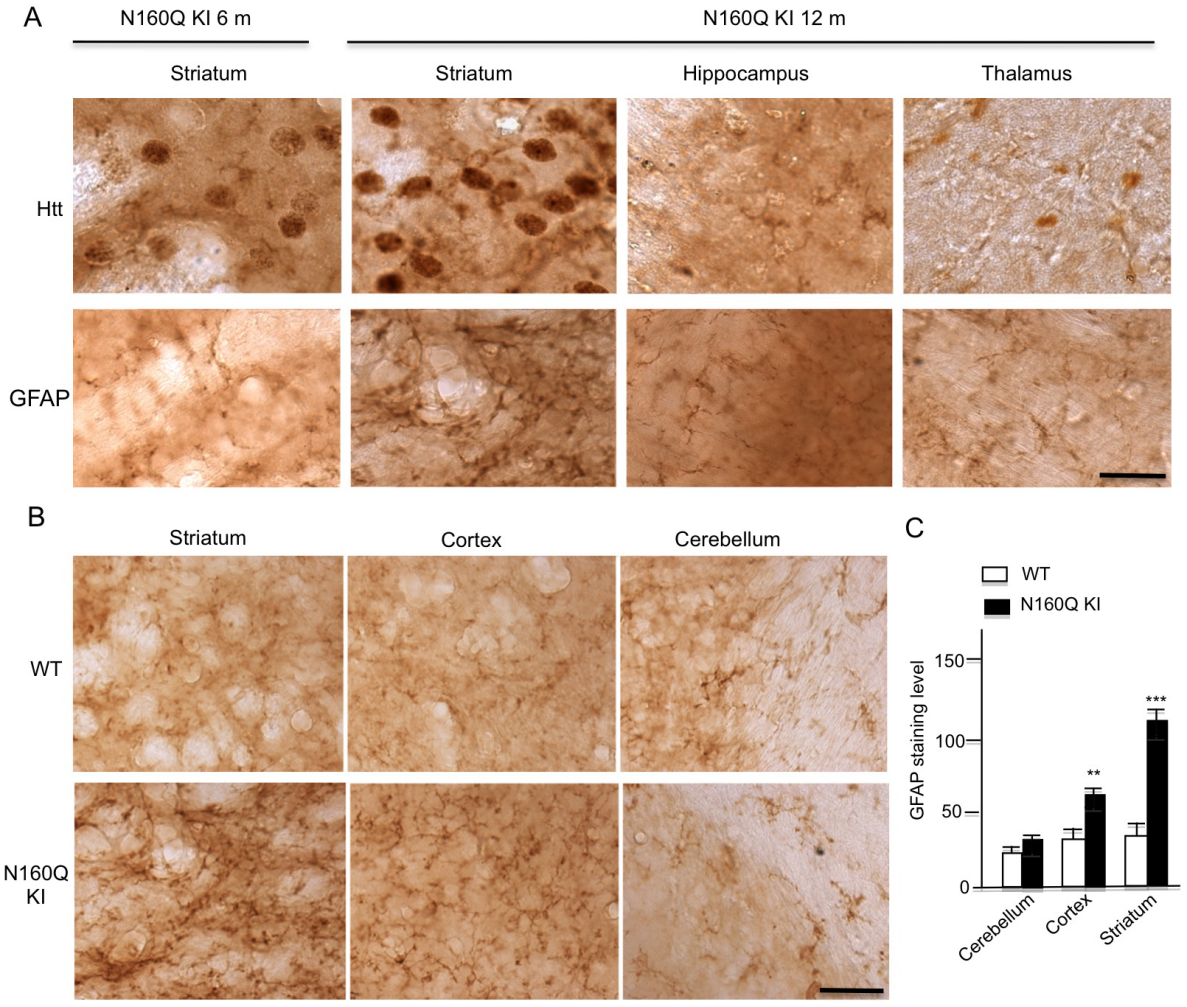
Age-dependent increase in reactive astrocytes in N160Q-KI mouse brains. **(A)** GFAP and HTT staining of N160Q-KI mice at 6 and 12 months of age. Note that increased GFAP occurs in the striatum and associates with the increased accumulation of mutant HTT in the striatum. HTT was stained by EM48 antibody. **(B)** More increased GFAP is seen in the striatum and cortex than cerebellum in 12-month-old N160Q-KI mice. (**C**) Quantification of the GFAP intensity in brain slices. The data are presented as mean±SE (n = 5–6 mice per group). ** P<0.01; *** P<0.001 compared with WT. Scale bars: 10 μm.

We know that HD KI mice show reactive astrocytes, an early neuronal injury event [24–27]. Examining N160Q KI mice also revealed reactive astrocytes, which are more abundant in the striatum in older mice (12 months) than young mice (6 months) (Fig 3A). Considering that N160Q preferentially accumulates in the striatum, we wondered whether the extent of reactive astrocytes is associated with the expression of N160Q. We used immunocytochemistry to examine the reactive astrocytes and quantified the relative extent of reactive astrocytes. By focusing on the striatum, cortex, and cerebellum in N160Q mice at 12 months of age, we found that the striatum shows the most abundant reactive astrocytes (Fig 3B). Using the same method as the one we reported recently to quantify reactive astrocytes [28], we verified that reactive astrocytes are the most abundant in the striatum and more abundant in the cortex than the cerebellum (Fig 3C). All these findings indicate that the accumulation of N160Q can cause age-dependent reactive astrocytes or early neuronal damage.

### Age-dependent neurological phenotypes in N160Q KI mice

**Fig 4:**
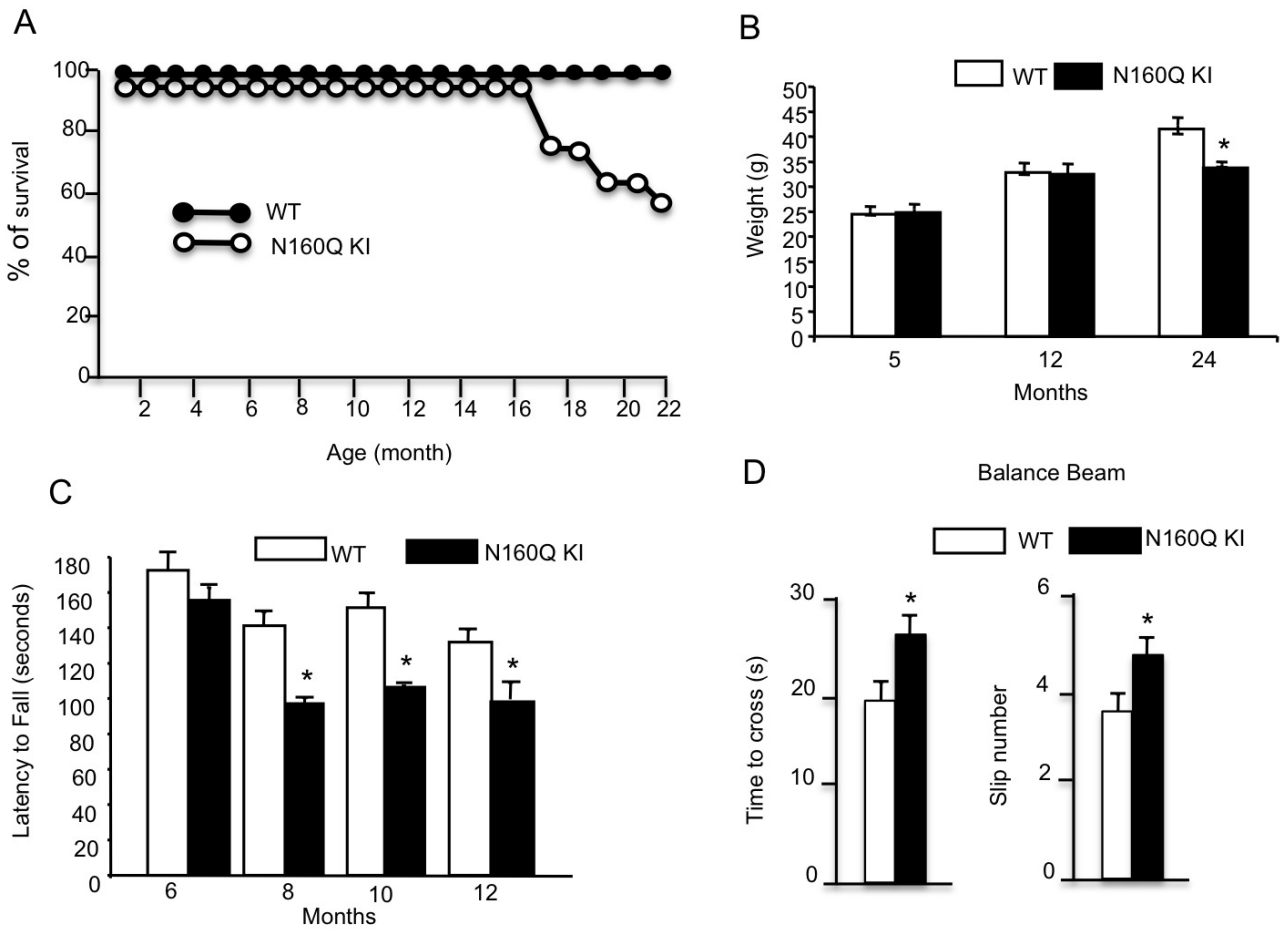
Age-dependent neurological phenotypes of N-terminal HTT KI mice. (**A**) The survival curve of N160Q-KI and wild type mice. (**B**) Body weight of N160Q KI mice. (**C**) Rotarod tests showing that motor deficits of N160Q KI mice increase with age. (**D**) Balance beam test showing a motor deficit of N160Q KI mice at 12 months of age. Age-matched WT mice (n = 19) and KI mice (n = 22) were examined. The data are presented as mean±SE. * P<0.05 compared with WT.

Slow and progressive development of neurological symptoms is the characteristic of HD KI mice [14–16]. Similarly, N160Q-KI mice also developed age-dependent neurological phenotypes. They grew normally as wild type mice from birth till 16 months, and some N160Q-KI mice then died earlier than wild type mice (Fig 4A); however, only the 24-month-old N160Q-KI mice experienced body weight loss (Fig 4B). Rotarod and balance beam tests are commonly used to assess the motor function of HD mice. We found that N160Q KI mice started to show impaired performance on the rotarod at 8 months compared with age-matched WT mice (Fig 4C). The balance beam test also revealed that N160Q-KI mice at 12 months of age took a longer time to cross the beam and slipped more frequently, which reflects a motor coordination defect (Fig 4D). Thus, like full-length HTT KI mice, N160Q-KI mice develop age-dependent neurological phenotypes, though these phenotypes are milder than those in HD mice that overexpress transgenic mutant HTT.

### Homozygous N160Q-KI mice are embryonic lethal

**Fig 5:**
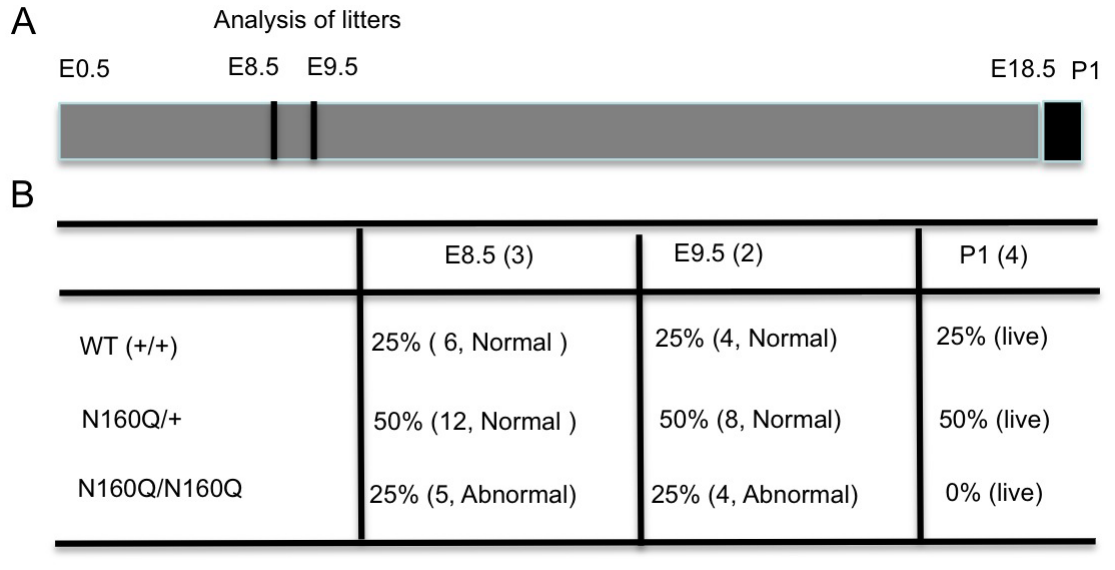
Embryonic lethality of homozygous N160Q-KI mice. **(A)** During mouse embryonic development, KI mouse embryos at embryonic day 8–9 were isolated for analysis of their morphology and genotypes, as well as numbers. **(B)** Summary of the numbers of normal and abnormal or re-absorbed embryos, their genotypes, and live pups from breeding N160Q KI mice.

Establishment of N160Q-KI mice allowed us to explore whether expression of the N-terminal HTT only could support early embryo development. Thus, we crossed heterozygous N160Q-KI mice and then genotyped all live newborn mice; however, we were unable to identify any live pups with the homozygous N160Q-KI genotype. This fact suggests that homozygous N160Q-KI embryos may not be able to develop to term. To confirm this, we collected embryos at day E8.5 and E9.5. Analysis of the embryos revealed that all healthy embryos were either the wild type or heterozygous N160Q-KI genotype. Some abnormal or absorbed embryos were identified as homozygous N160Q KI genotype at a 25% rate (Fig 5). Thus, unlike a large polyQ repeat (175Q) that does not affect the viability of newborn mice [29, 30] and transgenic full-length mutant HTT with expanded polyQ repeats that can rescue the Htt loss-mediated embryonic lethal phenotype [7, 8, 15], N-terminal HTT (1–208 amino acids) with an additional expanded polyQ repeat cannot support early embryonic development of mice when full-length mouse Htt is absent.

### HTT lacking the N-terminal region is protective against neuritic degeneration

**Fig 6:**
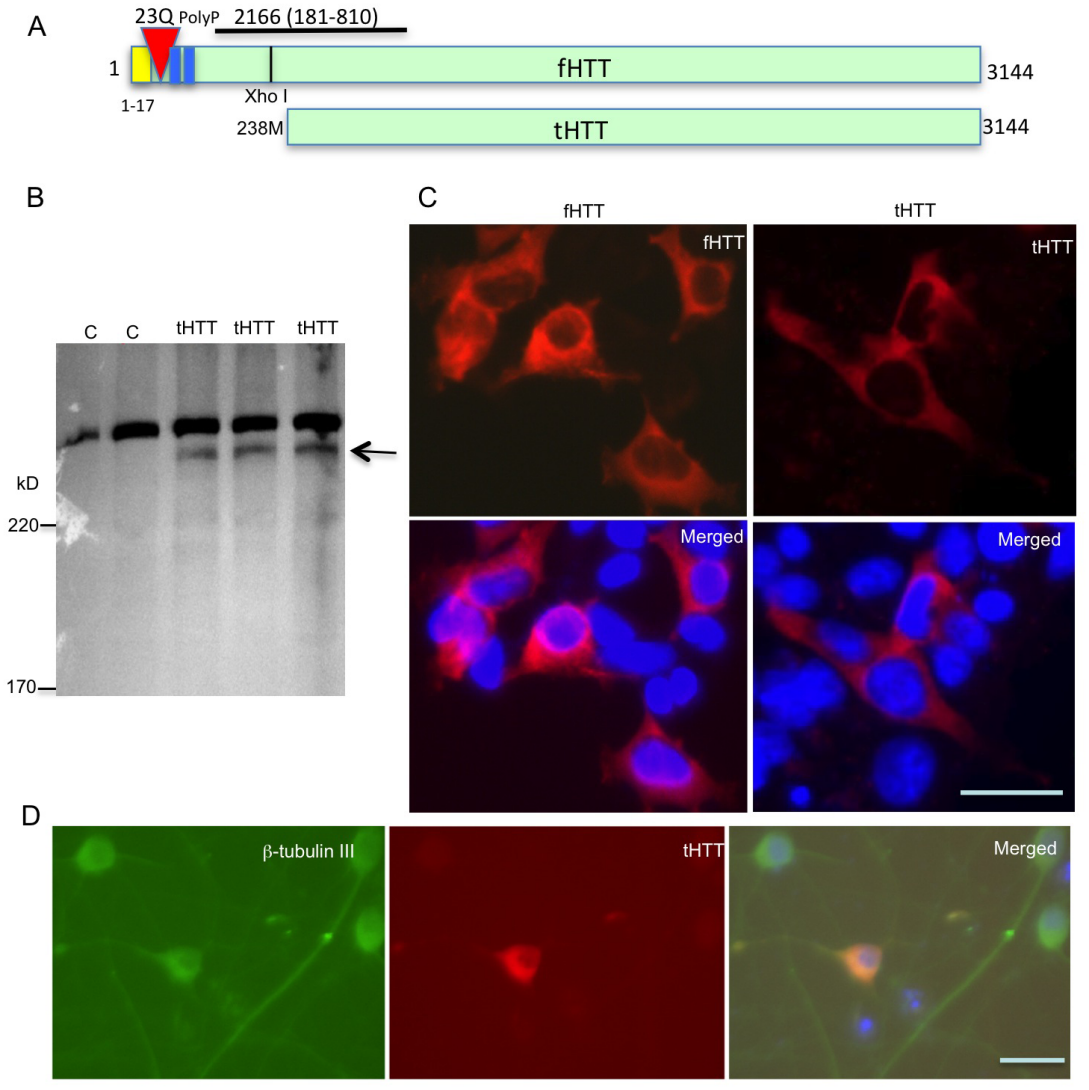
Expression of a truncated HTT in HEK293 cells. (**A**) DNA structure of the truncated HTT (tHTT) that lacks N-terminal 237 amino acids of HTT. Full-length HTT (fHTT) and their amino acids were also presented. (**B**) Western blotting revealed the expression of truncated HTT (arrow) in HEK293 cells. The band above tHTT is full-length HTT in HEK293 cells. C is untransfected cells. (**C**) Immunocytochemical analysis showing the cytoplasmic distribution as full-length HTT (fHTT) and truncated HTT (tHTT), suggesting that loss of the N-terminal fragment of HTT does not affect the normal cytoplasmic distribution of HTT. (**D**) Transfected primary neurons from mouse brain cortex also showing the cytoplasmic distribution of tHTT. Anti-β-tubulin III was used to label neuronal cells. Scale bars: 10 μm.

We know that HTT is important for brain development and the survival of developing neuronal cells [31–35], so the failure of N160Q-KI to rescue Htt loss-mediated embryonic lethality led us to investigate whether an HTT region other than the N-terminal region is important for neuronal development and survival. To this end, we generated a truncated HTT (tHTT) that lacks the first 237 amino acids, including the polyQ domain (Fig 6A). This truncated htt was expressed in HEK293 cells via transfection, and its expression was examined by western blotting with the antibody 2166, which was generated with HTT peptides containing 181–810 amino acids and reacts with the mouse Htt epitope at 421–434 amino acids [36] (Fig 6B). We also expressed normal full-length HTT containing 23Q as a control for comparison (Fig 6C). Immunocytochemical staining shows that tHTT, like full-length HTT (fHTT), is distributed predominantly in the cytoplasm (Fig 6C). Transfection of tHTT in cultured primate neurons from the mouse cortex also verified that tHTT is distributed in the cytoplasm (Fig 6D). Since normal HTT is predominantly present in the cytoplasm and an expanded polyQ stretch can lead to the nuclear accumulation of HTT and other cytoplasmic proteins [37], the cytoplasmic distribution of tHTT also suggests that this truncated HTT can perform the normal function of full-length HTT in the cytoplasm.

**Fig 7:**
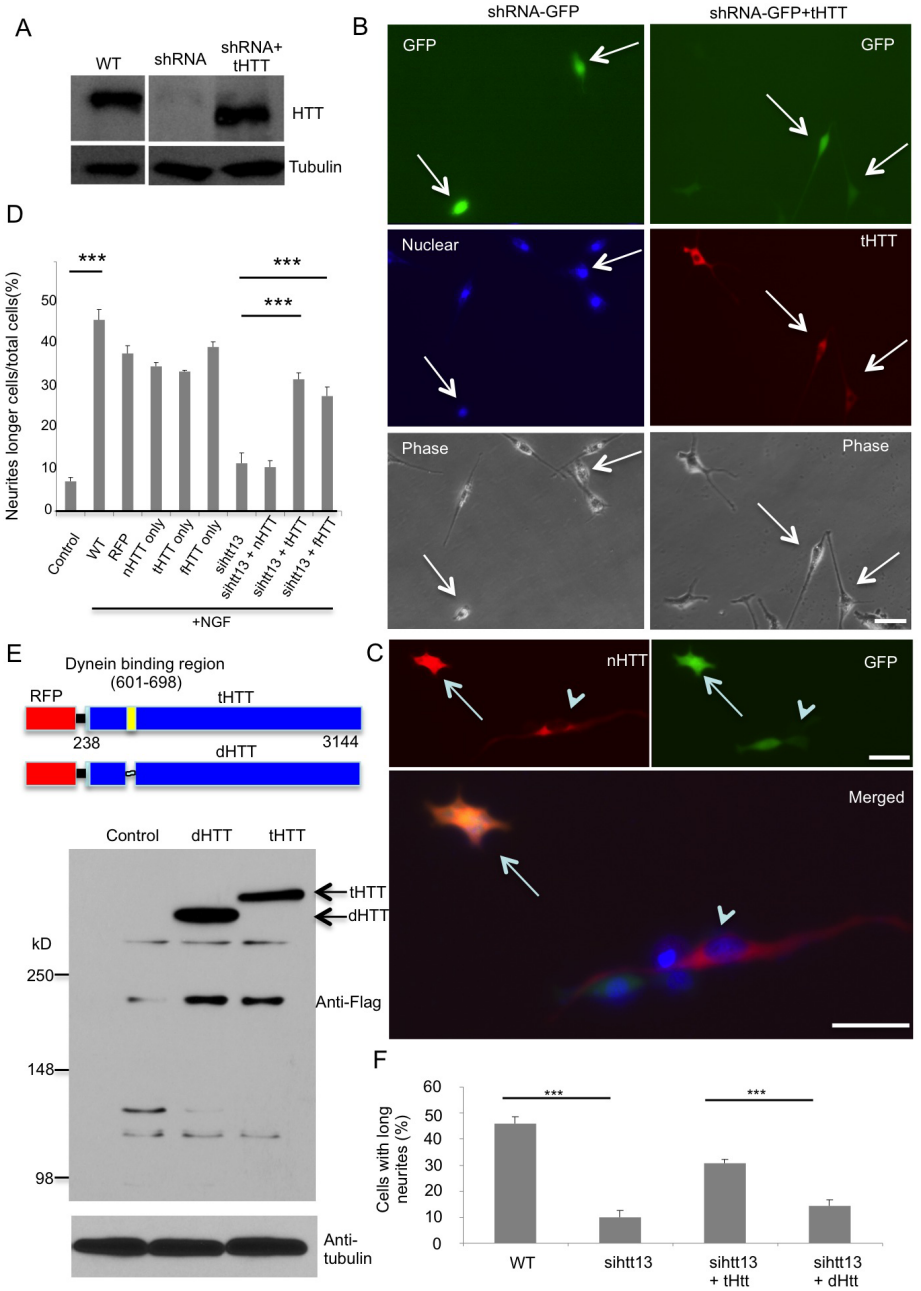
Truncated HTT lacking the N-terminal region is able to rescue neurite defects of PC12 cells. **(A)** Western blotting showing that HTT shRNA depleted Htt expression in PC12 cells, which were also transfected with RFP-tHTT. (**B**) Immunofluorescent staining of transfected PC12 cells showing RFP-tHTT can reduce the neurite extension defect in cells (red cells with arrows) that also express shRNA-GFP (green) to suppress endogenous Htt, whereas cells with shRNA-GFP alone (green cells in left panel) show defective neurite extension. The cells were treated with NGF (50 ng/ml) for 48 h to induce neurite extension. (**C**) Transfection of nHTT was unable to prevent Htt loss-mediated neuritic defect. Arrows indicate PC12 cell that expressed nHTT and also shRNA-GFP. Arrowheads indicate PC12 cells with long neurites that only expressed nHTT but not shRNA-GFP. In (B) and (C), scale bars: 10 μm. (**D**) Quantitation of the number of PC12 cells with neurites longer than two cell bodies. The data are presented as mean±SE (n = 500 cells per group). *** P<0.001. (**E**). Expression of the truncated HTT (tHTT) or mutant HTT (dHTT) that lacks the dynein-binding region in HEK293 cells. HTT is tagged with Flag and linked to RFP via P2A, a self-cleaving peptide. Western blotting with anti-Flag antibody reveals the expression of tHTT and dHTT. (**F**). Quantitation of the number of PC12 cells with neurites longer than two cell bodies after inhibiting Htt expression by shRNA and transfection with tHTT or dHTT. The data are presented as mean±SE (n = 500 cells per group). *** P<0.001.

Next, we examined whether tHTT is protective for neuronal cells that have depleted the expression of endogenous Htt. We first used PC12 cells because they can be efficiently transfected with shRNA to suppress Htt expression, and their neurite extension can be used to assess the effect of Htt deficiency on the neuronal differentiation property. We used shRNA, which is reported to efficiently inhibit HTT expression [38], to suppress Htt expression in the presence or absence of tHTT in PC12 cells (Fig 7A). This shRNA vector also expressed GFP, allowing us to identify cells in which Htt expression is inhibited, and tHTT expression could be identified by anti-HTT labeling. Double immunofluorescent staining showed that shRNA expression alone inhibited neurite extension of PC12 cells in response to NGF, while tHTT could prevent this neuritic extension defect (Fig 7B). However, expression of nHTT was unable to prevent the neuritic defect caused by the loss of Htt (Fig 7C). Quantification of the number of PC12 cells with neurites longer than two cell bodies confirmed that tHTT, but not nHTT, could reverse the neuritic extension defect caused by shRNA inhibition of Htt expression (Fig 7D). Neurite extension is largely dependent on intracellular trafficking, and htt is known to be involved in intracellular trafficking [1, 3]. Further, a previous study showed that HTT binds to dynein and acts in a complex along with dynactin [39–42] and Hap1 to facilitate vesicular transport, while HTT residues 600–698 are both necessary and sufficient for binding to dynein [39,40]. Thus, we generated a mutant HTT (dHTT) with deletion of the dynein-binding region (600–698 aa) and expressed it in PC12 cells, as well (Fig 7E). In this experiment, the transfected HTT is linked to RFP by a self-cleaving 2A peptide (P2A), whose self-cleavage in cells can separate RFP and transfected HTT. Thus, red fluorescent cells would also express transfected HTT and could be identified for examination. Compared with tHTT, dHTT was unable to prevent the neurite extension defect caused by HTT-shRNA (Fig 7F). This result supports the idea that the region in HTT important for binding to dynactin and intracellular trafficking is required for neuronal differentiation and development.

### Loss of Htt selectively affects cultured neurons but not glial cells

**Fig 8:**
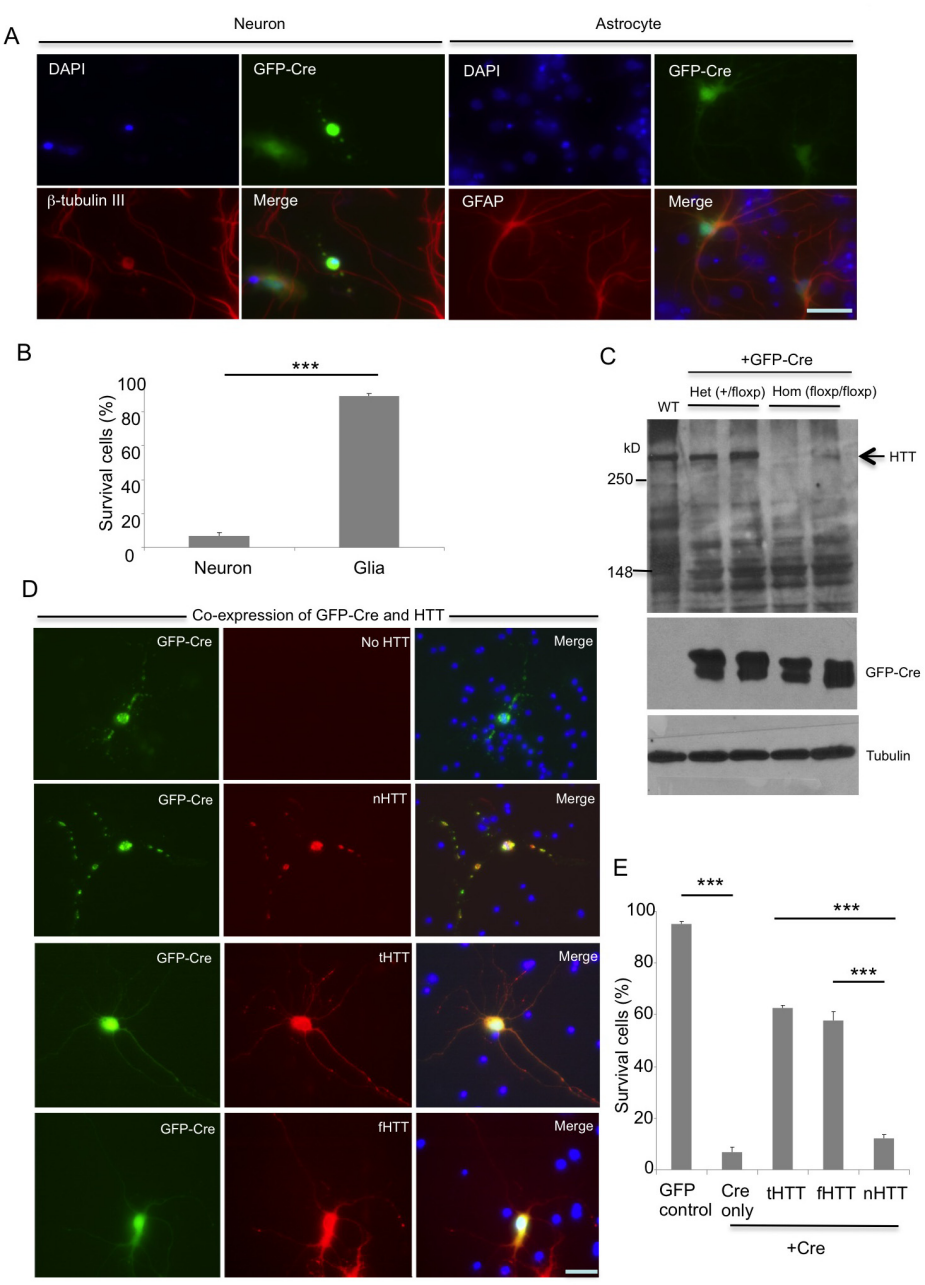
Truncated HTT lacking the N-terminal region can rescue the selective degeneration of cultured neurons that have deleted endogenous Htt. **(A)** Conditional Htt KO neuronal and glial cultures from floxed Htt mice were infected by adenoviral GFP-Cre to eliminate endogenous Htt expression in GFP-positive cells. Cultured striatal neurons and astrocytes were identified by antibodies to β-tubulin III and GFAP, respectively. Immunofluorescent staining shows that loss of Htt selectively causes neuronal, but not glial, degeneration. (**B**) Quantitative assessment of the surviving neurons and glial cells after the *Htt* gene is deleted by GFP-Cre expression. The control is cells infected with adenoviral-GFP without Cre. *** P<0.001. (**C**) Western blots with anti-HTT (2166) verified the depletion of Htt in primary cultures from the homozygous floxed Htt mouse brain cortex. (**D**) Transfection of nHTT, tHTT, or fHTT with 23Q into GFP-Cre-infected homozygous floxed Htt neurons demonstrating that tHTT and fHTT, but not nHTT (N-terminal HTT 1–208 amino acids), were able to rescue neurite degeneration caused by the loss of endogenous HTT. (**E**) Quantitative assessment of the surviving neurons after the endogenous *Htt* gene is deleted by GFP-Cre and transfection with different HTT forms. Adenoviral-GFP expression served as a control. *** P<0.001. Scale bars, (A and D): 10 μm.

To further test the protective effect of tHTT in primary neuronal cells, we cultured neurons from conditional Htt knockout mice in which the mouse *Htt* gene is flanked by loxP and can be deleted upon Cre expression [43]. After cultured striatal cells were infected with adenoviral GFP-Cre, cells showing GFP signals should have the *Htt* gene depleted. We found that neuronal cells displayed fragmented neurites after the *Htt* gene is depleted via Cre expression. However, there was no morphological change in cultured astrocytes that also express GFP-Cre (Fig 8A). We then counted the percentage of neurons and astrocytes with GFP-Cre relative to cells that were infected by adenoviral GFP without Cre and found there was a significant reduction in the surviving neurons after the *Htt* gene is deleted by Cre (Fig 8B). Western blotting was also performed to validate the depletion of Htt expression in primary cell cultures from the brain of the homozygous floxed Htt mice (Fig 8C). Next, we expressed nHTT, tHTT, and fHTT in conditional Htt knockout neurons that express GFP-Cre. N-terminal HTT (nHTT) was unable to rescue the neuritic degeneration of neurons after deletion of the *Htt* gene, while fHTT and tHTT were capable of rescuing this neuritic degeneration, which was demonstrated by both double immunofluorescent staining (Fig 8D) and quantitative analysis of the numbers of neurons with long neurites (Fig 8E).

**Fig 9:**
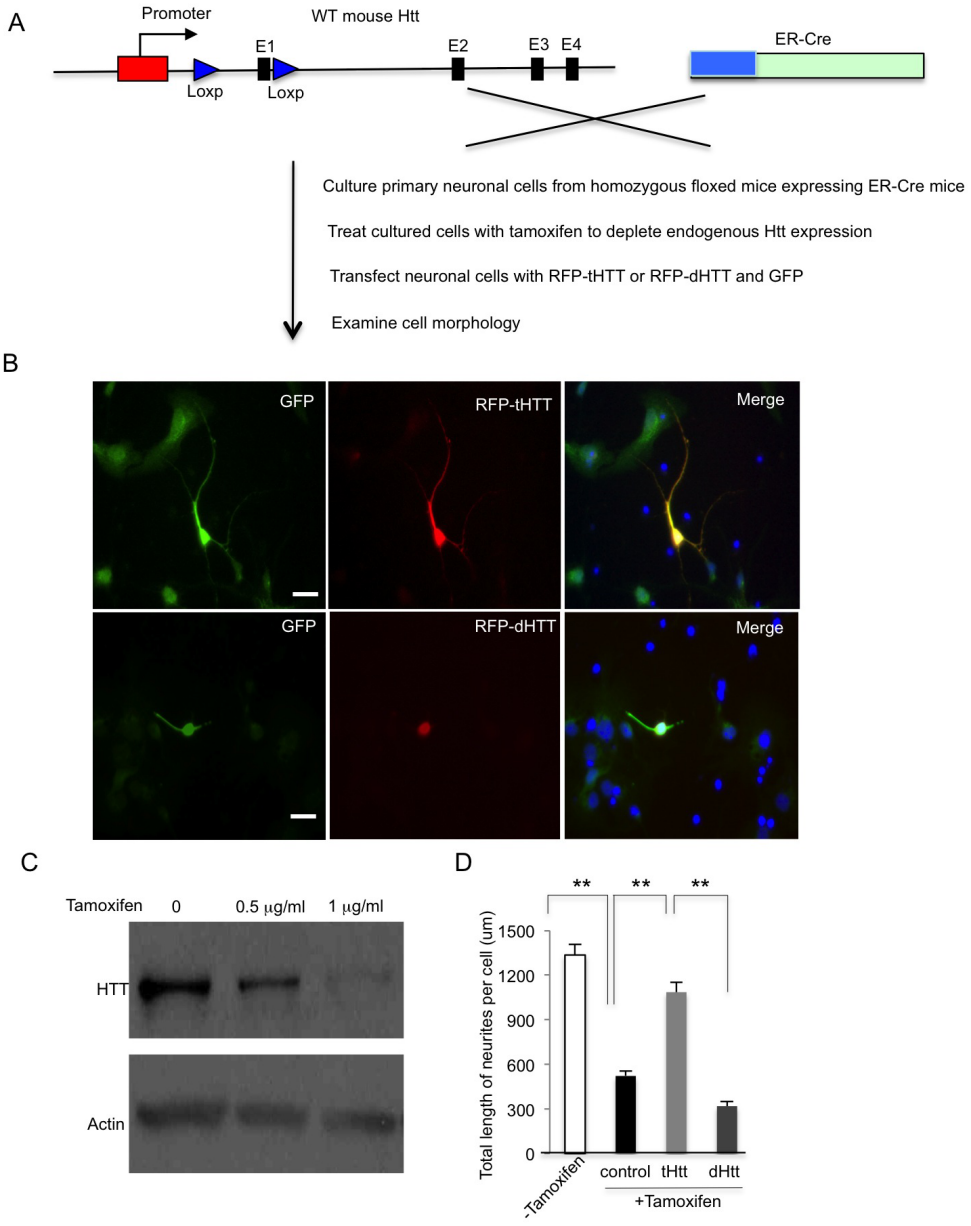
Truncated HTT lacking the N-terminal region can rescue the degeneration of cultured neurons by inducible depletion of the *HTT* gene. (**A**) Schematic strategy to generate inducible HTT knockout neurons by tamoxifen. (**B**) HTT-inducible knockout neurons were transfected with tHTT or dHTT. Immunostaining showing that tHTT can increase neuronal processes, while dHTT is unable to protect against neuritic degeneration. The DIV 3 cortical neurons from floxed Htt mice with Cre were co-transfected with RFP-tHTT or RFP-dHTT and GFP. GFP immunostaining clearly reveals the processes of transfected cells. After 24 h transfection, the cells were treated with tamoxifen (1 g/ml) for 72 h to deplete endogenous mouse Htt. Immunofluorescent staining reveals the long processes of neurons expressing RFP-tHTT, but not RFP-dHTT, when endogenous mouse Htt is depleted. Scale bars: 10 m. (**C**) Western blotting confirming a dose-dependent depletion of Htt by tamoxifen in cultured cortical neuronal cells. (**D**) Quantitative measurement of total length of neurites per cell in each group after the endogenous *Htt* gene is deleted by tamoxifen. ** P<0.01.

Although we used adenoviral GFP as a control to compare with adenoviral GFP-Cre to identify the specific effect of Cre-mediated Htt depletion, tamoxifen-induced Cre expression can also lead to gene depletion without overexpressing viral Cre. Thus, we crossed conditional Htt KO mice to transgenic mice that express ER-Cre, which can be activated by tamoxifen to enter the nucleus and delete the floxed genes by loxP sites (Fig 9A). The primary cortical neurons were isolated from the crossed mice and co-transfected with RFP-tHTT or RFP-dHTT and GFP, as GFP immunofluorescent staining could clearly reveal the processes of transfected neurons. The transfected cells were then treated with tamoxifen to deplete endogenous mouse Htt. We found that RFP-tHTT, but not RFP-dHTT that lacks the dynein-binding region, could rescue the defective processes of neuronal cells caused by the depletion of endogenous mouse Htt (Fig 9B). Western blotting confirmed that mouse Htt is depleted by tamoxifen treatment (Fig 9C), and counting the numbers of transfected cells also showed that expression of tHTT, but not dHTT, was able to rescue neuritic degeneration caused by the loss of mouse Htt (Fig 9D). Taken together, loss of Htt appears to affect the differentiation and neurites of developing neurons, and this adverse effect can be alleviated by expression of tHTT without the N-terminal region.

## Discussion

The function of the N-terminal region containing the polyQ domain is an important focus of investigation. This is because the first 17 amino acids of HTT are well conserved in different species, and modifying them can alter HTT toxicity [19, 20, 22] and deletion of N17 can facilitate the accumulation of mutant Htt in the nucleus of BACHD transgenic mice [20]. However, although polyQ expansion in N-terminal HTT can cause age-dependent neurodegeneration, this expansion does not affect early development in most HD patients. Consistently, mice expressing mutant HTT with large polyQ repeats show no abnormalities in embryonic and early development. Also, transgenic full-length human HTT with expanded polyQ repeats can rescue the embryonic lethal phenotype in mice [8, 44]. Deletion of the N-terminal 17 amino acids, polyQ, or the proline-rich domain does not affect mouse development [11, 12]. Moreover, the N-terminal region of HTT (<500 amino acids) interacts with a large number of partners [45, 46]. All these findings underscore the importance of understanding the role of the N-terminal region of HTT in addition to the polyQ domain.

We know that HTT is important for brain development and neuronal survival [29]. Conditional knockout of Htt in the postnatal period by expression of Cre under the CamKII promoter is reported to also lead to the degeneration of a limited number of neurons and testis cells in mice [43]. By studying N160Q KI mice, our findings provide evidence that N-terminal HTT is unable to support embryonic development but can cause age-dependent neurological symptoms. In our new KI mouse model, the mouse Htt exon 1 is replaced with human N-terminal HTT (1–208 aa). Because long polyQ repeats can cause juvenile HD cases, it may be that a large polyQ repeat can affect the function of HTT in early development. Our studies cannot exclude the possibility that 160Q affects the function of N-terminal HTT during early embryo development so that no homozygous N160Q KI mice were born. Thus, a rigorous control would be to establish knock-in mice that express N-terminal HTT with a normal polyQ repeat to examine whether N-terminal HTT is required for embryonic development.

The milder phenotypes of N160Q KI mice can be explained by multiple possibilities. We found that N160Q expression is lower than the full-length mutant HTT in heterozygous KI mice that also express one allele of the mutant HTT gene. Insertion of the Neo selection cassette in intron 1 that follows exon 1 may affect the splicing and/or stability of mRNA encoded by the exon 1 DNAs, thus resulting in the low level of N160Q transcripts in the mouse brain. Exon 1 mutant HTT can be produced by aberrant splicing and contributes to the pathology of HD KI mice [13]. However, our N160Q KI mice express a cDNA that is unable to produce exon 1 via aberrant splicing, such that the lower level of exon 1 mutant HTT in N160Q KI mice may also account for the less severe phenotypes. Furthermore, the size of N-terminal HTT fragments may determine the severity and nature of HD neuropathology, as smaller N-terminal mutant HTT fragments are more prone to misfolding and aggregation. Our N160Q KI mice express a longer N-terminal HTT fragment (1–208 aa) than R6/2 mice, which express the smaller exon 1 mutant HTT (1–67 aa) at a level similar to endogenous HTT and show much more severe and progressive neurological phenotypes [47]. Nevertheless, N160Q-KI mice show typical age-dependent HTT aggregation in the brain and late-onset neurological symptoms. The neurological symptoms in N160Q-KI mice are milder than in those HD mice that overexpress mutant HTT.

N160Q-KI mice also show preferential accumulation of mutant HTT in striatal neurons, in the same manner as full-length HTT KI mice [14–16]. This preferential accumulation mirrors the vulnerability of striatal neurons in HD patient brains. The interesting finding in our study is that the first 208 amino acids of HTT are sufficient to mediate the selective accumulation of mutant HTT in striatal neurons, suggesting that proteolytic N-terminal fragments of mutant HTT can also selectively accumulate in striatal neurons. This finding will spur further studies to explore the critical region in the N-terminal HTT sequences and mechanisms that underlie the selective striatal neuronal degeneration in HD.

Heterozygous Htt KO mice with the expression of Htt at 50% of wild type Htt are known to develop and grow normally. The failure of N160Q to rescue embryonic lethality could be due to its low expression level. However, since early lethality can be bypassed in hypomorphic Htt mutant mice in which Htt expression is about one third of wild type levels [35] and since the N160Q level in heterozygous mice is at 51–75% of full-length mutant HTT in heterozygous F140Q KI mice, the death of homozygous N160Q embryos is more likely due to the loss of functional HTT. The transfection data that overexpressed N-terminal HTT cannot prevent the HTT loss-mediated degeneration of cultured neurons also suggest that N-terminal HTT (1–208 aa) may lack the functional domain(s) that are essential for neuronal survival. In support of this idea, our findings suggest that a truncated HTT lacking the N-terminal region is able to prevent neuronal degeneration caused by the loss of HTT in developing neurons. We have used three different approaches to reduce Htt expression via shRNA, GFP-Cre expression, and tamoxifen-induced depletion. All these methods can efficiently reduce Htt expression in cultured neuronal cells, allowing us to show that truncated HTT without the N-terminal region is protective against HTT loss-mediated degeneration of developing neurons. In addition, the lack of a dynein-binding domain in tHTT diminished the protective effect, also supporting the role of HTT in intracellular trafficking. It should be pointed out that HTT is a multifaceted protein that can functionally regulate many cellular functions. For example, tHTT also contains the domain that may be involved in autophagic function [48, 49]. Multiple functional domains in HTT could play broad or cell type-specific roles in different types of cellular functions. Neuritic extension is critically dependent on intracellular trafficking, and depleting the region critical for intracellular trafficking may have a more dramatic impact on neurite elongation. Whether and how other functional domains in tHTT are important for early development and neuronal survival remains to be investigated.

Our findings also uncover important therapeutic implications for HD. The modification of N17 amino acids is found to alter HD pathology in transgenic mice [19, 20, 22]. The modification relies on the phosphorylation and ubiquitination of HTT and reveals some druggable targets. However, specific drugs that can selectively inhibit HTT toxicity without other side effects remain to be developed. Anti-sense and shRNA have become powerful approaches to suppress HTT expression [50–52]. Because HTT is essential for embryonic development, considerable efforts have gone in to developing specific inhibitors that may only affect mutant but not wild type HTT [48]. Our recent study shows that depletion of Htt in adult neurons is non-deleterious and transgenic HTT without N-terminal region is functionable [53]. Although whether loss of HTT can affect neuronal function in adult brain remains to be investigated, it is likely that removal of N-terminal region of mutant HTT is therapeutically beneficial. Recently developed new technology, such as CRISPR-Cas9, would allow achievement of this deletion. Because CRISPR-Cas9 can selectively delete the targeted gene in postmitotic neuronal cells [54–57] and its targeting can permanently delete the gene, removal of N-terminal HTT could be a more efficient therapeutic approach than those that require continuous administration of drugs or chemicals for disease treatment.

## Materials and Methods

### Ethics statement

All procedures were performed in accordance with the NIH and U.S. Public Health Service’s Guide for the Care and Use of Laboratory Animals and were approved by the Institutional Animal Care and Use Committee at Emory University with an approved IACUC protocol (2002557), which is accredited by the American Association for Accreditation of Laboratory Care (AAALC). All of the care for the animals is consistent with standard operating procedures, and all efforts were made to minimize suffering.

### Reagents and antibodies

Full-length HTT in pEBVHis vector and N-terminal HTT in PRK vectors were established in our previous studies [58, 59]. To generate truncated HTT (tHTT) with the deletion of N-terminal HTT (1–237 aa), the truncated HTT was released from full-length HTT plasmids by XhoI and NotI digestion, and inserted into pEBVHis vector. The DNAs for RFP, P2A-Flag, and HTT (238–600 aa) were generated by PCR. Primers used were as follows: RFP (forward, 5’- CC ATC GAT GGC ACC ATG GCC TCC TCC GAG AAC GTC ATC-3’; reverse, 5’- CC ATC GAT CTC GAG CAG GAA CAG GTG GTG GCG G-3’); P2A-Flag (forward, 5’- TCC GCT CGA GGG AGG AAG CGG AGC TAC TAA CTT C-3’; reverse, 5’- CCG GAA TTC TTT GTC ATC GTC ATC CTT GTA GTC TTT G-3’); HTT (238–600 aa) (forward, 5’- CCG GAA TTC ATG GCT TCT TTT GGC AAT TTT GC-3’; reverse, 5’- TCC CCC GGG GGG CTG TCC AAT CTG CAG-3’). The digested DNA products were cloned to pRK5 vector to generate dHTT with deletion of the dynein-binding region (601–698 aa).

The anti-huntingtin antibodies (rabbit EM48 and mouse mEM48) were produced previously in our laboratory [60]. The mouse antibody 1C2 was purchased from Millipore (Temecula, CA). The mouse anti-γ-tubulin antibody was purchased from Sigma-Aldrich (St. Louis, MO) and used at a 1:50,000 dilution. Additional antibodies used were those against GAPDH (Ambion), HTT (2166, Millipore), **β**-tubulin III (Abcam), and Flag (Abcam). Adenoviral GFP-Cre was obtained from Vector Biolabs. The shRNA plasmids used to knock down HTT (siHTT13) and GFP (siGFP) were obtained from Dr. Nicole Déglon’s lab [38].

### Animals

All animal procedures were approved by the Institutional Animal Care and Use Committee of Emory University. Full-length mutant HTT knock-in (CAG140) mice were provided by Dr. Michael Levine at UCLA [61] and were maintained in the animal facility at Emory University in accordance with institutional guidelines. N208-160Q knock-in mice (N160Q KI) were generated by targeting a human *HTT* cDNA encoding the first 208 amino acids with 160CAG repeats to exon1 of the mouse *Htt* gene. A transcriptional stop codon was added to the 3’ end of *HTT* cDNA in the targeting vector to ensure the expression of truncated HTT. This targeting vector contains the PGK-neomycin resistance gene that is flanked by two loxP sites and was transfected via electroporation into SV/129-derived embryonic stem (ES) cells. Positive ES cells containing the targeted vector were identified by genomic DNA PCR and southern blotting, and two clones of these cells were then injected by the Emory Transgenic Facility into C57B1/6J blastocysts to generate chimeric mice. Heterozygous N160Q KI mice were then produced by mating male chimeric mice with female wild-type C57B1/6J mice. PCR genotyping of N208-143Q mice used the following primers (forward 5'-GGC CTT CGA GTC CCT CAA GTC CTT CCA G-3' and reverse 5'-TGC TGC TGA GGC TGA GGA AGC TGA G-3'). Conditional Htt KO mice in which the mouse *Htt* gene is flanked by loxP were provided by Scott Zeitlin at University of Virginia [43]. To generate inducible Htt knockout mice, we crossed conditional Htt KO mice with B6.Cg-Tg (CAG-cre/Esr1)5Amc/J; The Jackson Laboratory) transgenic mice that express tamoxifen-inducible Cre throughout the body to generate homozygous Htt-floxed mice that also express Cre.

### Cell culture

HEK293 cells were cultured in Dulbecco’s modified Eagle’s medium supplemented with 10% FBS, 100 **μ** g/mL penicillin, 100 units/mL streptomycin, and 250 **μ** g/μL fungizone amphotericin B. Cells were incubated at 37°C in 5% CO_2_. At a confluency of 70%, the cells were transfected with 1–2 **μ** g/well (6-well plate) or 0.5–1 **μ** g/well (12-well plate) of DNA and lipofectamine (Invitrogen) for 48 h. Brains of postnatal (day 1–3) murine pups were used for culturing cortical astrocytes. Following dissection, cortex was subjected to 0.3 mg/ml papain digestion. Cell suspension flew through 70- **μ** m nylon cell strainers (Fisher). Cells were plated onto Petri dishes, and culture medium was replaced 24 h later and once every 3 days thereafter. Microglia and oligodendrocytes were removed from cultures by shaking at DIV14. Remaining cells were detached with 0.25% trypsin and plated for the following experiments. For cortical neuron cultures, cortical neurons were prepared from postnatal day 0 murine pups. Cortex was digested with 0.3 mg/ml papain. Cell suspension was filtered through 40- **μ** m nylon cell strainers (Fisher) to remove debris. Neurons were plated at 1×10^6^ on poly-D-lysine-coated 6-well plates, and cultured in Neurobasal-A medium supplemented with B27 and glutamine (Invitrogen). Half the culture medium was changed with fresh medium every 3 days. PC12 cells were cultured in Dulbecco's modified Eagle's medium supplemented with 10% horse serum, 5% fetal bovine serum, 100 U/ml penicillin, 100 **μ** g/ml streptomycin, and 0.25 **μ** g/ml amphotericin B. To evaluate neurite outgrowth, PC12 cells were treated with NGF (100 ng/ml). For transfection, cells were transfected with plasmid DNA using Lipofectamine 2000 (2mg/mL from Invitrogen) for 6 h in serum-free medium.

### Immunocytochemistry

Mice were anesthetized and perfused intracardially with phosphate-buffered saline (PBS, pH 7.2) for 30 s, followed by 4% paraformaldehyde in 0.1 M phosphate buffer (PB) at pH 7.2. Brains were removed, cryoprotected in 30% sucrose at 4°C, and sectioned at 40 μm using a freezing microtome. Free-floating sections were in 4% paraformaldehyde in 0.1 M phosphate buffer for 10 min and preblocked in 4% normal goat serum in PBS, 0.1% Triton X, and then incubated with mEM48 antibody at 4°C for 48 h. The immunoreactive product was visualized with the Avidin–Biotin Complex kit (Vector ABC Elite, Burlingame, CA, USA).

Cultured cells were fixed with 4% paraformaldehyde for 15 min, and then blocked for 1 h with 3% BSA and 0.2% triton X-100 in 1X PBS. Cells were incubated overnight with primary antibodies in 3% BSA in 1X PBS. The nucleus was visualized using Hoechst staining (Molecular Probes) at a dilution of 1:5000. Fluorescent images were obtained using a Zeiss microscope (Axiovert 200 MOT) with a digital camera (Hamamatsu Orca-100) and OpenLAB software (Improvision Inc).

### Histological quantification

We used a quantitative method described in our previous study [28] to quantify the degenerated cultured neurons or PC12 cells with elongated processes; images were collected from at least 5 different randomly selected areas per brain section or plate. To measure GFAP immunostaining intensity, ImageJ software was used. Colored images obtained with a 40X objective, NA 0.95, were first converted to 8-bit black-and-white images. The “Threshold” function was used to adjust the background to highlight GFAP-specific staining. The same threshold was applied to all images analyzed. Finally, the “Measure” function was used to quantify GFAP staining intensity in each image. Each group with 5 to 8 images were examined. Transfected PC12 cells were also imaged randomly to quantify cells with normally elongated neurites that were longer than two cell bodies, and at least 500 transfected cells per group were used for statistical analysis.

### Mouse behavioral analysis

Mouse body weight was measured twice every month, and survival was monitored regularly. The motor function of the mice was assessed with the rotarod test (Rotamex, Columbus Instruments). Mice were trained on the rotarod at 5 RPM for 5 min for 3 consecutive days. After training, the mice were tested for 3 consecutive days, 3 trials per day. The rotarod gradually accelerated to 40 RPM over a 5-min period. Latency to fall was recorded for each trial. The balance beam test was run using a 0.6 cm thick meter stick suspended from a platform on both sides by metal grips. The total running distance was roughly 0.8 m. There was a bright light at the starting point and a dark box at the endpoint. Prior to data collection, each mouse was trained for 3 consecutive days with 3 runs per day.

### Western blot and RT-PCR analyses

Mouse brain tissues or harvested cells were lysed in ice-cold RIPA buffer (50 mM Tris pH 8.0, 150 mM NaCl, 1 mM EDTA pH 8.0, 1 mM EGTA pH 8.0, 0.1% SDS, 0.5% DOC, and 1% Triton X-100) containing Halt protease inhibitor cocktail (Thermo Scientific) and phosphatase inhibitors. The lysates were incubated on ice for 30 min, sonicated, and centrifuged at top speed for 10 min. The supernatants were subjected to SDS-PAGE. The proteins on the gel were transferred to a nitrocellulose membrane, which was then blocked with 5% milk/PBS for 1 h at room temperature. The blot was incubated with primary antibodies in 3% BSA/PBS overnight at 4°C. After 3 washes in PBS, the blot was incubated with HRP-conjugated secondary antibodies in 5% milk/PBS for 1 h at room temperature. After 3 washes in PBS, ECL Prime (GE Healthcare) was then used to detect immunoreactive bands on the blot.

For RT-PCR, total RNA was isolated from the mouse brain tissues using the RNeasy Lipid Tissue Mini Kit (Qiagen). Reverse transcription reactions were performed with 1.5 **μ** g of total RNA using the Superscript III First-Strand Synthesis System (Invitrogen, 18080–051). cDNA (100 ng) was combined with 10 **μ** l SYBR Select Master Mix (Applied Biosystems, 4472908) and 1 **μ** l of each primer in a 20- **μ** l reaction. The reaction was performed in the Eppendorf, Realplex Mastercycler thermocycler using primers (primers specific for human *HTT* were 459S 5’-GCCGCCTCCTCAGCTTCCTCAG-3’ and 565A 5’-GTCGGTGCAGCGGCTCCTC-3’ [62]. The relative values of PCR results were normalized by GAPDH levels prior to calculations.

### Statistical analysis

All data are expressed as mean±SEM. The statistical significance was determined by two-tailed Student’s t-tests or two-way ANOVA, followed when appropriate by post hoc t-tests using GraphPad Prism 5.0 software. A value of p<0.05 was considered statistically significant.
